# Predicting malaria risk considering vector control interventions under climate change scenarios

**DOI:** 10.1038/s41598-024-52724-x

**Published:** 2024-01-29

**Authors:** Margaux L. Sadoine, Kate Zinszer, Ying Liu, Philippe Gachon, Michel Fournier, Guillaume Dueymes, Grant Dorsey, Ana Llerena, Jane Frances Namuganga, Bouchra Nasri, Audrey Smargiassi

**Affiliations:** 1 School of Public Health, Université de Montréal, Montreal, Quebec Canada; 2 Center for Public Health Research, Université de Montréal, Montreal, Quebec Canada; 3 ESCER (Étude et Simulation du Climat à l’Échelle Régionale) Centre, Université du Québec à Montréal, Montreal, Quebec Canada; 4 Department of Public Health, Montreal Regional, Montreal, Quebec Canada; 5 University of California San Francisco, San Francisco, USA; 6 Department of Earth and Atmospheric Sciences, Université du Québec à Montréal, Montreal, Quebec Canada; 7 Infectious Diseases Research Collaboration, Kampala, Uganda

**Keywords:** Malaria, Environmental impact

## Abstract

Many studies have projected malaria risks with climate change scenarios by modelling one or two environmental variables and without the consideration of malaria control interventions. We aimed to predict the risk of malaria with climate change considering the influence of rainfall, humidity, temperatures, vegetation, and vector control interventions (indoor residual spraying (IRS) and long-lasting insecticidal nets (LLIN)). We used negative binomial models based on weekly malaria data from six facility-based surveillance sites in Uganda from 2010–2018, to estimate associations between malaria, environmental variables and interventions, accounting for the non-linearity of environmental variables. Associations were applied to future climate scenarios to predict malaria distribution using an ensemble of Regional Climate Models under two Representative Concentration Pathways (RCP4.5 and RCP8.5). Predictions including interaction effects between environmental variables and interventions were also explored. The results showed upward trends in the annual malaria cases by 25% to 30% by 2050s in the absence of intervention but there was great variability in the predictions (historical vs RCP 4.5 medians [Min–Max]: 16,785 [9,902–74,382] vs 21,289 [11,796–70,606]). The combination of IRS and LLIN, IRS alone, and LLIN alone would contribute to reducing the malaria burden by 76%, 63% and 35% respectively. Similar conclusions were drawn from the predictions of the models with and without interactions between environmental factors and interventions, suggesting that the interactions have no added value for the predictions. The results highlight the need for maintaining vector control interventions for malaria prevention and control in the context of climate change given the potential public health and economic implications of increasing malaria in Uganda.

## Introduction

Vector-borne diseases, often transmitted by hematophagous insects, are among the major concerns associated with climate change. Malaria is a life-threatening disease that accounted for over 241 million cases and an estimated 619,000 deaths in 2021^1^. Among vector-borne diseases, it is regarded as one of the most sensitive to climate change^2^. The intricate relationship between specific environmental conditions such as temperature and malaria has been extensively documented and quantified. An increase in temperature leads to faster development of *Anopheles* mosquito and a higher frequency of its blood meal uptake^3,4^, with optimal temperatures for transmission estimated between 25 and 27 °C^5,6^. Rainfall creates breeding sites necessary for larval development and increased humidity influences mosquito longevity and parasite development^7^. Extreme rainfall, however, may wash out any mosquito larvae in these pools or aquatic environments, thereby decreasing mosquito and parasite populations^8^. With changing climatic patterns, exposure to malaria will likely change in Sub-Saharan Africa^9,10^ and it is projected that a shift of the dominant malaria species (*Anopheles gambiae* and *Anopheles arabiensis*) may occur in several regions in Sub-Saharan Africa by 2055^11^.

Several studies have projected the risk of malaria according to climate change scenarios on regional scales^11–16^. While climatic effects on malaria transmission are evident, projecting future malaria risks is a complex endeavor. Almost all these studies focus on a few meteorological parameters and omit anthropogenic factors that could influence future malaria occurrence^10^. Although studying the isolated potential impact of climate is certainly informative, not considering the influence of malaria control interventions is an important limitation, given their significant role in the fight against malaria^17,18^. Yet, very few studies have considered the influence of interventions in their predictions^19,20^.

In Uganda, malaria is endemic in 95% of the territory with an estimate of 12.6 million cases in 2021^1^. In this country, malaria control relies primarily on indoor residual spraying of insecticide (IRS) programs targeting epidemic areas and nationwide campaigns of long-lasting insecticides nets (LLINs). Uganda was the first country to introduce mass distribution of LLINs and had one of the highest coverage rates with 93% of households reportedly owning at least one LLIN in 2021^21^. Despite sustained efforts in vector control since the 2000s, malaria remains a leading cause of morbidity and mortality in Uganda, accounting for 30–50% of outpatient visits, 15–20% of all hospital admissions, and up to 20% of all hospital deaths^22^. In this context, the implications of climate change are especially concerning. Rising temperatures and changes in precipitation patterns could result in changing transmission intensity. In addition, the 5% of the territory located in the highlands of Uganda where temperatures have historically been less favourable to mosquitoes and parasitic development, could experience an emergence of the disease; such an emergence has been previously associated with large-scale morbidity and mortality in other East African highlands^23,24^. This study aims to estimate the future risk of malaria in six regions of Uganda by 2050 based on different climate change scenarios. Given that very few studies have projected the risk of malaria while accounting for vector control interventions, particular emphasis was placed on considering interventions such as long-lasting insecticidal nets (LLINs) and indoor residual spraying (IRS), together with various environmental determinants such as precipitation, temperature, specific humidity, and vegetation. In addition, the non-linear relationships between environmental factors and malaria control intervention were explored.

## Results

### Climate projections

The evolution of climate for the historical (1975–2004) and the future periods (2036–2065) for both RCP4.5 and RCP8.5 scenarios are presented in Figure S1 from Supplementary file 1. The distribution of meteorological factors for the RCMs average under historical and future periods are also presented in Table S1 from Supplementary file 1. Minimum and maximum temperatures and specific humidity are forecasted to be greater in 2036–2065 compared to the historical period for both RCP scenarios and more pronounced for RCP8.5. By the period centred on 2050 under RCP8.5, mean annual changes (based on weekly data) will be approximately + 2.5 °C for both minimum and maximum temperatures, and + 0.0015 kg kg^−1^ for specific humidity under RCP8.5. Precipitation patterns will remain relatively unchanged in the future for both RCPs in Uganda.

### Climate models and regression model assessment

A description of the results of the spatial and temporal bias analyzes as well as the evaluation of the regression models is provided in Supplementary file 2. The results and interpretation of the temporal bias analyzes with the Taylor diagrams are presented in Figures S2 to S5 from Supplementary file 1, the spatial bias maps are shown in Figures S6 to S9 from Supplementary file 1, and errors associated with prediction from the regression models are presented in Table S8 from Supplementary file 1.

### Malaria incident cases 2036–2065

**Figure 1:**
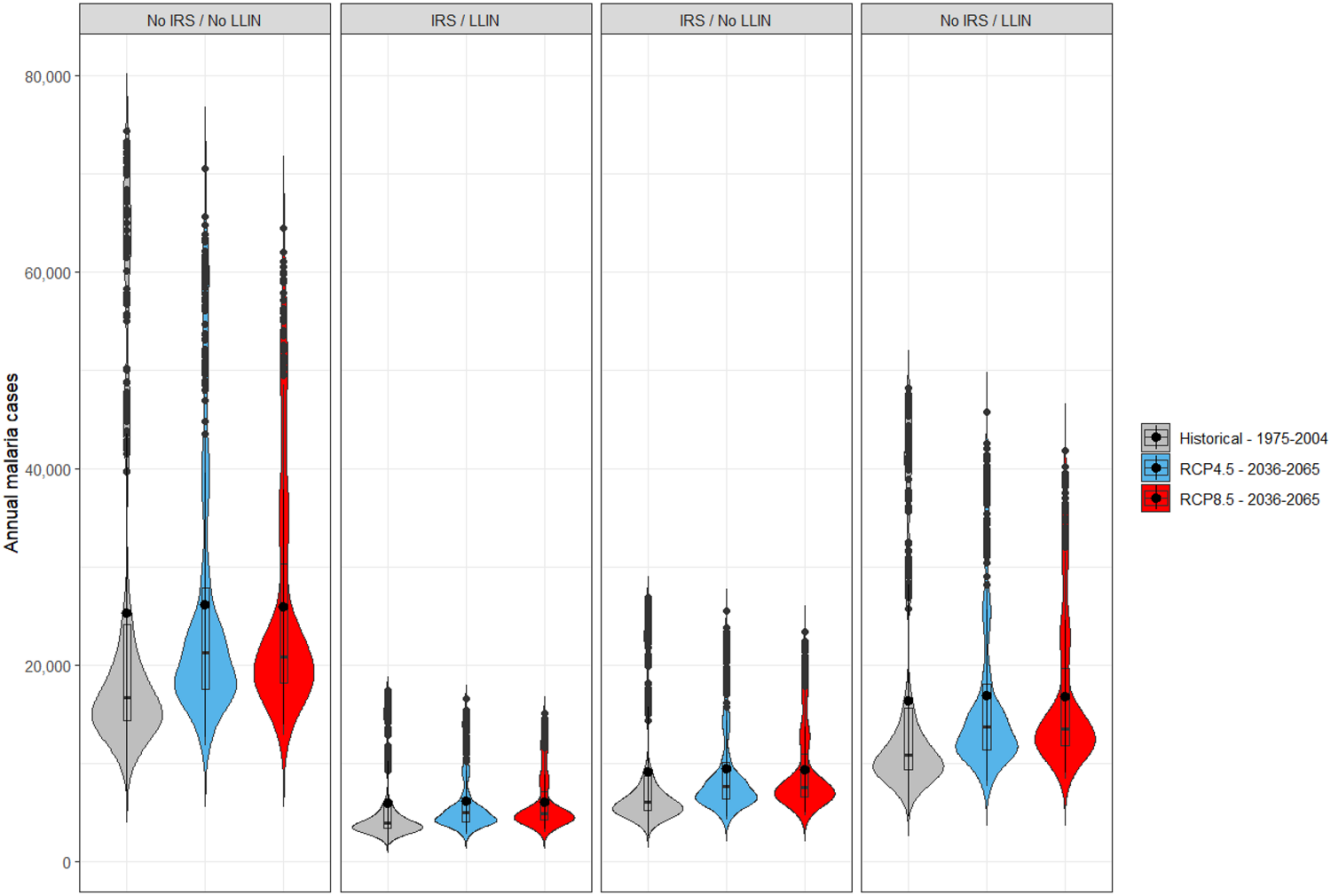
Distribution of annual sum of weekly malaria cases predicted for the past (1975–2004) and the future (2036–2065) periods for the negative binomial GLM without interaction between environmental variables and interventions; The mean of the distributions is represented by the black dots; (N = 30 years*14 climate models*4 interventions*3 periods).

The distributions of the average number of malaria cases, visits, EVI, and income used for the prediction are presented in Table S2 from Supplementary file 1. The distribution of the annual sum of weekly predicted cases is presented in Fig. 1 and Table S3 from Supplementary file 1 for the model without interaction between environmental variables and interventions, and Figure S7 and Table S4 from Supplementary file 1 for the model with interactions.

Results showed an upward trend in malaria cases by 2050s but with large variability in the predictions (median [Min–Max]: 16,785 [9,902–74,382] for the reference period versus 21,289 [11,796–70,606] under RCP 4.5) (Fig. 1 and Table S3 from Supplementary file 1). Similar results were obtained with the model with interaction (Table S4 from Supplementary file 1).

It was shown with the models that the combination of bednets (LLINs) and indoor spraying (IRS) reduced the annual malaria cases, with IRS providing greater protection than that offered by LLINs alone (Fig. 1). For the model without interaction, the comparison of the medians of each intervention to no intervention in each period (historical, RCP4.5, RCP8.5) showed a reduction in annual malaria cases by 76.5%, 63.8% and 35.1% with the two vector control interventions, IRS alone and LLIN alone, respectively (Table S3 from Supplementary file 1). Based on the relative difference of the medians with and without intervention, the influence of interventions in the future is comparable to that of the past (model without interaction: −35% to −76% cases in the past and the future; model with interaction: −26% to −72% cases in the past and −17% to −69% in the future) (Tables S3 and S4 from Supplementary file 1).

**Figure 2:**
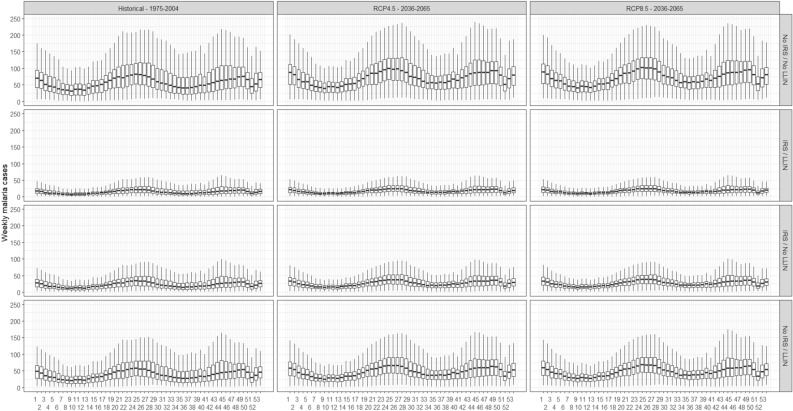
Distribution of weekly malaria cases predicted for the past (1975–2004) and the future (2036–2065) periods from the binomial negative GLM without interaction; (outliers removed and N = 53 weeks*6 health centers*14 climate models*4 interventions*30 years*3 periods).

The distributions of weekly malaria cases predicted from the model without interaction are shown in Fig. 2. These results suggest no change in malaria seasonality by 2050 for both RCP scenarios compared to the baseline. Medians, maximum, and minimum weekly predictions with 95% confidence intervals are also presented in Figures S8 and S9 from Supplementary file 1 for the model without interaction and with interaction, respectively. When considering the confidence intervals of the weekly predictions, a larger variability of the predictions was observed.

## Discussion

Our study is the first to have estimated the potential impact of climate change on malaria risk in six regions of Uganda by 2050, considering (i) various environmental factors and their non-linear relationship with malaria, (ii) the influence of vector control interventions, and (iii) interactions between environmental variables and interventions, according to an empirical-statistical approach. Although our results showed an increase in the median yearly cases of malaria of 25% to 30% in the absence of intervention in Uganda by 2050, there are considerable variabilities in the predictions. However, the predictions in this study clearly highlight the beneficial impact of vector control interventions in reducing the malaria burden, particularly if combining indoor residual spraying (IRS) and long-lasting insecticidal nets (LLIN). The comparison of the regression models with and without interactions between environmental variables and interventions leads to similar conclusions about the possible effects of climate and interventions. Given these results and the larger variability of the predictions of the model with interactions, we found no added value in considering interactions between environmental predictors and interventions for predicting malaria risks under climate change scenarios.

Several studies have predicted an increase in malaria risks in Africa according to different climate change scenarios^11–16^, particularly in the highlands where the environmental conditions and the limited immunity of highland residents could favor a rapid emergence and potential epidemic of malaria^16,25^. These studies were based on mathematical models that analyze the dynamics of the transmission between hosts and vectors and/or the influence of the environment on the different stages of the development of mosquitoes. These studies also used emissions from the Special Report on Emissions Scenarios (SRES)^11,13,15,16^, GCMs^11–13^ and varied duration periods for predictions (10 to 30 years). We used instead a statistical model to predict malaria cases over a 30 year-period, based on regional climate models (RCM) and the representative concentration pathways RCP4.5 and RCP 8.5. To the best of our knowledge, only a few studies^26–28^ have used statistical modelling to predict malaria risks with climate change and malaria control interventions have almost never been considered^19,20^. The study by van Lieshout et al.^20^ which used mathematical modelling, GCMs and previous emission scenarios (SRES A1FI, A2, B1 and B2), considered contemporary vulnerability to malaria (based on the absence or presence of malaria control and their level of funding, the strength of health infrastructure) to establish future adaptation capacities to change in malaria risks. The authors projected that the greatest additional population at-risk of malaria by 2080 will be observed in three regions including that of East Africa (and Uganda). This prediction, however, was based on a low level of malaria endemicity in the region, with little investment and implementation of malaria control measures, which is not representative of the Ugandan context^20^. The study by Moukam Kakmeni et al.^19^, also based on mathematical modelling, estimated that by considering fictional intervention scenarios which would influence certain vector parameters (mosquito biting rate, vector competence, adult mosquito mortality rate, and the probability that mosquito eggs survive to become adults), a significant reduction in malaria transmission is likely to occur by 2050 in most regions of Africa and particularly in Kenya^19^.

Despite a different methodological approach, our findings showed that interventions would likely have a substantial impact on malaria occurrence in the context of climate change, reducing the annual number of malaria cases upwards to 76% in the presence of both vector control interventions (IRS and LLINs) in Uganda. Moreover, our predictions tend to align with the possible increase of 25 to 30% in malaria cases with climate change in a scenario with no control measures. Such an increase not only poses significant public health concerns but also economic implications for Uganda, whose GDP loss was nearly $50 million USD in 2003 due to malaria morbidity^29^.

Our results must however be interpreted with caution given the very high level of uncertainty of the predictions. Extreme malaria predictions were associated with certain models that showed notable differences in the distribution of some climatic variables (i.e. more precipitation and lower maximum temperatures) compared to models that predicted fewer malaria cases. Climate models can be biased for various reasons such as due to simplified representations of complex processes. Differences between models highlight the importance of considering an ensemble of climate models to account for their uncertainties and to provide more robust estimates. Nonetheless, the large variability in past and future predictions makes it challenging to draw decisive conclusions on the future risk of malaria with climate change.

There are many limitations and sources of uncertainty in the inputs and methodologies used in this study that need to be acknowledged. First, the absence of observed malaria cases over the historical period to validate the past predictions (1975–2004) is an important limitation of our work. Second, we used an explanatory model to estimate malaria risk, assuming that the exposure–response association will not change in the future. This assumption has many limitations given the possibility of insecticide vector resistance, changes in vector species composition, socioeconomic changes, and demographic changes, which are all elements that could influence and shape the malaria burden in the future. Third, the number of visits, vegetation, and income in the future were imputed by their average observed between 2010–2018, which is also a limitation of our analysis, as these imputed values likely influenced the predictions. Fourth, a predictive model could have estimated more accurately the number of cases than an explanatory model; however, they require more observation points and the relationships with predictors from such models are more difficult to interpret. Fifth, we used the number of clinic visits as the offset of the model, thus modeling the number of malaria-positive diagnosis among clinic visits. Several limitations are associated with this approach. The ratio of visits with malaria positive diagnosis may not be a good proxy for a population-based incidence rate and only reflect malaria burden among individuals seeking care, the number of which may vary depending on different factors (health centers capacities, perception of the quality of care, etc.). Furthermore, the proportionality between incident cases and the number of visits induced by the offset can be problematic for predicting future malaria cases. If the proportion of malaria positive diagnosis to clinic visits is high, the upper limit of the confidence interval estimated by the model may then exceed the projected number of visits. Despite these limits, the total number of clinic visits was used as the offset as it provided the most accurate and readily available estimate of the population at-risk given that population-based administration division estimates are outdated^30^ and importantly do not necessarily reflect the catchment areas^31^. Sixth, we have developed our regression models based on certain indicators such as cumulative precipitations but other relevant variables could be considered when predicting malaria risk such as precipitations intensity. Seventh, the prediction errors of our regression models were relatively large (40 cases per week for an average of 73). However, as the over-optimism estimation was very low, the risk of overfitting is negligible, suggesting that if the model was used with new data, similar predictive accuracy would be obtained. Eighth, although we used two contemporary greenhouse gas emissions scenarios (intermediate – RCP.5 and pessimistic – RCP8.5) and a set of 14 different climate projections to reduce the uncertainty inherent to each model, it is not guaranteed that the actual future scenario will be captured among the simulated conditions. Finally, the projections were established from a pooled model (combining data from six subregions of Uganda) which could therefore mask subregional disparities in the effects of interventions and environmental factors; however similar predictions were obtained with the pooled and the subregional-specific models in our previous study^32^. Nonetheless, establishing projections at the subregional level with higher resolution (ex., 0.11°) RCMs’ simulations available through the updated CORDEX CMIP6 project (with the more recent Shared Socioeconomic Pathways – SSP- scenarios used in the last AR6 IPCC report^33^), would improve predictions and be relevant to better inform decision-makers on the future risk of malaria.

## Conclusion

Our study results predict an increase of malaria cases in the future and also show that the continued investments in LLINs and IRS will significantly reduce the occurrence of malaria in Uganda according to different climate change scenarios. These findings support the importance of including the potential influence of malaria control measures when predicting the effect of climate change on malaria risks. It also emphasizes the importance of sustained malaria control and their funding and can offer policymakers a lens to assess the financial ramifications of malaria burden in the context of climate change.

## Data and methods

### Data and statistical models

In this study, we used a statistical model developed previously^32^ to estimate the number of malaria cases. Briefly, a generalized linear model based on a negative binomial distribution was used to estimate the association between malaria incident cases and environmental factors (rainfall, minimum and maximum temperature, specific humidity, and vegetation). The model was based on weekly malaria incident cases in the general population (0 to 70 years), registered between 2010–2018, from six malaria reference centres (MRC) that are part of a malaria surveillance program at health facilities throughout Uganda (dependent variable). The model's offset was the weekly total number of visits to each MRC and a fixed effect for the MRC was applied. Daily environmental data were obtained from remote sensed sources—Africa Rainfall Climatology Version 2^34^ for rainfall, MODIS^35^ for the Enhanced Vegetation Index (EVI), and ERA5 reanalyses^36^ for minimum and maximum temperature and specific humidity (representing the mass of water vapour per kilogram of moist air). Four-month moving averages for minimum and maximum temperatures, specific humidity, and EVI, and the four-month cumulative rainfall preceding each week of malaria counts at health facilities were used to analyze associations between environmental variables and malaria. The nonlinearity of the associations between environmental variables and malaria incident cases was considered using natural cubic splines with three knots placed at the 10th, 50th and 90th percentiles^37^. The model controlled for the household’s average monthly income and vector control interventions (long-lasting insecticidal nets (LLINs) and indoor residual spraying (IRS) coverage). All regions received two universal net distribution campaigns during 2010–2018 and we considered their effect over a 2-year period (based on survival data from the literature^38–42^). As for the IRS, only two regions received continuous rounds of spraying over defined periods. Given this, we considered coverage from the month following the first round until the end of the residual efficacy period of the last round (residual efficacy period derived from^43^). Given that there were significant interaction effects between certain environmental variables and vector control interventions, we also considered a model with interactions for predictions. Significant interactions were identified between IRS and minimum temperature/ specific humidity/ EVI, while all interactions between LLINs and environmental variables were statistically significant, with the exception of specific humidity. Equations and estimates of the regression models without interaction and with interactions are presented in Tables S5, S6 and S7, respectively.

Accuracy of the regressions models was estimated by comparing the distribution of the weekly observed cases to the predicted cases by our models over the period 2010–2018.

Finally, since regression models are generated to provide the best fit for the available data, there was a risk that the accuracy measures of the models are over-optimistic and would not translate on future datasets. A more realistic estimate of the performance of the model on new datasets can be obtained with cross-validation. An optimistic assessment of the predictive ability of the regression models was performed using a cross-validation approach^44^. Details of the calculation of these accuracy measures corrected for "over-optimism" are presented in Table S8.

### Intervention and climate scenarios for prediction

The regression models were used to predict malaria incident cases under the future climate centred on 2050 (i.e., period 2036–2065). Predictions were conducted according to four intervention scenarios: (i) the absence of vector control interventions, (ii) the simultaneous presence of LLINs and IRS, (iii) the presence of IRS only, and (iv) the presence of LLINs only. The future number of visits, income, and vegetation were variables in the model for which no data were available for the future. Therefore, we used the average number of visits and EVI per season and site over the period 2010–2018, as well as the average income per site to predict the number of future malaria cases. For the projection of the climate of 2050 (i.e., 2036 to 2065 period), we used four regional climate models (RCMs) simulations driven by global climate models (GCMs) from the 5th phase of the Coupled Model Intercomparison Project (CMIP5)^45^. These GCM-driven RCMs were considered under the representative concentration pathway (RCP) 4.5 and 8.5^46^, deemed as the most realistic representation of global warming (ref for this statement as you say deemed). The RCP are greenhouse gas emission scenarios that are used in climate models to simulate the future climate response. The RCP4.5 scenario represents a medium greenhouse gas emission scenario in which efforts are being made to limit emissions although with few climate change policies being implemented. RCP8.5 scenario represents a pessimistic projection with high levels of greenhouse gas concentrations being emitted and assumes no implementation of climate change policies^46^.

All the models used for climate projections in this study are listed in Table 1. The RCMs are produced within the defined COordinated Regional climate Downscaling EXperiment over Africa (CORDEX-Africa^47^; http://wcrp-cordex.ipsl.jussieu.fr) and provide daily data at the 0.44° (approximately 50 km) horizontal resolution. The CORDEX-Africa data used in this work were obtained from the CORDEX website (https://cordex.org/domains/region-5-africa/). Daily data from each GCM-driven RCMs were transformed into four-month moving averages preceding each week for the period 2036–2065 and used directly in the statistical model to project future weekly malaria cases. Given that the time window for historical climate data availability depended on the climate models and ending at most in 2005, the reference period for the present climate was therefore chosen over a 30-year period common to all models, and corresponding to 1975–2004.

### Bias assessment

To identify potential temporal biases within the climate models, comparisons were made with both the GCM-driven RCMs and ERA-interim (reanalyses of observations) driven RCMs (ERAINT) using the Taylor Diagrams. This method offers a statistical analysis of the degree of pattern correspondence between the modelled data and observed data, through their correlation, their root-mean-square difference, and the ratio of their variances^48^. The comparison was done over the largest overlapping time-period between datasets, corresponding to 1989–2004. The ERA5 datasets were degraded to a resolution of 0.44° to match the GCM-driven RCMs’ resolution in order to be used as reference data in Taylor Diagrams. Spatial biases were evaluated for the same period by calculating absolute biases between the GCM-driven RCMs’ and ERA5 for maximum and minimum temperatures, and relative biases for precipitation and specific humidity. These calculations were carried out using the open-source program CDO (Climate Data Operator)^49^.

### Analysis tools

The analyses were performed using R program version 4.2.2. Taylor Diagrams were produced with the *openair* package^50^ and the weekly number of malaria cases under climate change was predicted using the function predict of *car* package^51^. The 95% confidence interval of the weekly malaria predictions was calculated by multiplying their standard error by 1.96. Finally, the annual cases were calculated from the sum of the weekly predicted cases. Spatial bias maps were produced with Python3 (https://www.python.org/download/releases/3.0/) using the Cartopy^52^ and Matplotlib^53^ libraries (Table 1).

**Table 1:** Reanalysed (ERA-interim driven) and Simulated (GCM-driven) Regional Climate Models (RCMs).

RCM	Institute	Driving model
CCLM-4-8-17^54^	Climate Limited-area Modelling Community (CLM-Community)	ERA-interim
		CNRM-CERFACS-CNRM-CM5
		ICHEC-EC_EARTH
HIRHAM5-v2^55^	Danish Meteorological Institute	ERA-interim
		ICHEC-EC-EARTH
RCA4-v1^56^	Swedish Meteorological and Hydrological Institute (SMHI)	ERA-interim
		CCCma-CanESM2
		CNRM-CERFACS-CNRM-CM5
		CSIRO-QCCCE-CSIRO-Mk3-6-0
		ICHEC-EC-EARTH
		IPSL-IPSL-CM5A-MR
		MIROC-MIROC5
		MOHC-HadGEM2-ES
		MPI-M-MPI-ESM-LR
		NCC-NorESM1-M
		NOAA-GFDL-GFDL-ESM2M
RACMO22T^57^	Royal Netherlands Meteorological Institute (KNMI)	ERA-interim
		ICHEC-EC-EARTH

### Ethics approval

This study uses secondary data from health facilities routinely collected on outpatients and deidentified. Ethical approval from the School of Public Health of the Université de Montréal Ethics Committee and complies with to the Tri-Council Policy Statement: Ethical Conduct for Research Involving Humans.

### Supplementary Information

Supplementary Information 1.

Supplementary Information 2.

## Data Availability

Climate data was accessed through https://cordex.org/domains/region-5-africa/. To obtain raw data for these analyses, please contact the corresponding author.
