# Supplementary material for: Predicting malaria risk considering vector control interventions under climate change scenarios
Source: Sci Rep. 2024 Jan 29;14:2430. doi: 10.1038/s41598-024-52724-x (PMC10824718; doi:10.1038/s41598-024-52724-x)
Supplement: Supplementary file 1 — Supplementary Information 1. [file 41598_2024_52724_MOESM1_ESM.docx]

**Supplementary material – file 1**

**-**

Projecting malaria risk considering vector control interventions under climate change scenarios -

Margaux L. Sadoine, Kate Zinszer, Ying Liu, Philippe Gachon, Michel Fournier, Guillaume Dueymes, Grant Dorsey, Ana Llerena, Jane Frances Namuganga, Bouchra Nasri, and Audrey Smargiassi

**Table S1.** Annual distributions of meteorological factors from 14 Regional Climate Models (RCMs) under historical (1975-2004) and future (2036-2065) periods

| **Period** | **Descriptive statistics** | **Rainfall (mm)** | **Maximum temperature (°C)** | **Minimum temperature (°C)** | **Specific humidity (kg.kg-1)** |
| --- | --- | --- | --- | --- | --- |
| **Historical**  **(1975-2004)** | Mean (SD) | 288 (238) | 26.3 (3.59) | 15.8 (2.62) | 0.0111 (0.00211) |
|  | Median [Min, Max] | 239 [0, 1820] | 26.9 [17.1, 36.7] | 16.0 [6.78, 22.4] | 0.0113 [0.00339, 0.0170] |
| **Future – RCP 4.5**  **(2036-2065)** | Mean (SD) | 279 (218) | 28.3 (3.52) | 17.7 (2.52) | 0.0122 (0.00226) |
|  | Median [Min, Max] | 241 [0, 1840] | 28.6 [18.8, 38.1] | 17.9 [8.96, 24.8] | 0.0124 [0.00407, 0.0186] |
| **Future – RCP 8.5**  **(2036-2065)** | Mean (SD) | 290 (236) | 28.8 (3.62) | 18.3 (2.61) | 0.0126 (0.00233) |
|  | Median [Min, Max] | 242 [0, 1810] | 29.1 [19.3, 39.1] | 18.5 [9.43, 25.9] | 0.0129 [0.00406, 0.0197] |

**Table S2.** Distribution of the weekly number of both malaria cases and visits, and enhanced vegetation index (EVI) averaged per season and malaria reference center (MRC), and the average income per site over the period 2010-2018 ^[[1]](#footnote-1)^

| **Descriptive statistics** | **Average number of malaria cases at the MRC** | **Average number of visits at the MRC** | **Average Enhanced vegetation index (EVI)** | **Average income**  **(Ugandan Shilling)** |
| --- | --- | --- | --- | --- |
| Mean (SD) | 73.3 (17.9) | 426 (123) | 0.407 (0.0420) | 309 000 (102 000) |
| Median [Min, Max] | 72.2 [26.1, 98.5] | 400 [134, 757] | 0.410 [0.300, 0.480] | 350 000 [142 000, 420 000] |

**Table S3.** Annual malaria cases (based on weekly sums) predicted over the reference period and for 2050s for the model without interaction

| **Period** | **Intervention** | **Median** | **Min – Max** | **Absolute difference between the median predictions with interventions compared to the median without intervention, in each period** | **Relative difference between the median predictions with intervention compared to the median without intervention, in each period (%)** |
| --- | --- | --- | --- | --- | --- |
|  |  |  |  |  |  |
| Historical  (1975-2004) | No IRS / No LLIN | 16 785 | 9 902 – 74 382 |  |  |
|  | IRS / LLIN | 3 946 | 2 328 – 17 487 | 12 839 | -76.49 |
|  | IRS / No LLIN | 6 084 | 3 589 – 26 965 | 10 701 | -63.75 |
|  | No IRS / LLIN | 10 885 | 6 422 – 48 239 | 5 900 | -35.15 |
| Future – RCP 4.5  (2036-2065) | No IRS / No LLIN | 21 289 | 11 796 – 70 606 |  |  |
|  | IRS / LLIN | 5 005 | 2 773 – 16 600 | 16 284 | -76.49 |
|  | IRS / No LLIN | 7 718 | 4 276 – 25 596 | 13 571 | -63.75 |
|  | No IRS / LLIN | 13 807 | 7 650 – 45 790 | 7 482 | -35.15 |
| Future – RCP 8.5  (2036-2065) | No IRS / No LLIN | 20 910 | 12 948 – 64 524 |  |  |
|  | IRS / LLIN | 4 916 | 3 044 – 15 170 | 15 994 | -76.49 |
|  | IRS / No LLIN | 7 580 | 4 694 – 23 391 | 13 330 | -63.75 |
|  | No IRS / LLIN | 13 561 | 8 397 – 41 846 | 7 349 | -35.15 |

LLIN = long-lasting insecticidal nets; IRS = Indoor residual spraying

**Table S4.** Annual malaria cases (based on weekly sums) predicted over the reference period and for 2050s for the model with interactions

| **Period** | **Intervention** | **Median** | **Min – Max** | **Absolute difference between the median predictions with interventions compared to the median without intervention, in each period** | **Relative difference between the median predictions with interventions compared to the median without intervention, in each period (%)** |
| --- | --- | --- | --- | --- | --- |
|  |  |  |  |  |  |
| Historical  (1975-2004) | No IRS / No LLIN | 14 918 | 7 994 – 146 869 |  |  |
|  | IRS / LLIN | 4 103 | 1 7997 – 12 778 | 10 815 | -72.49 |
|  | IRS / No LLIN | 5 849 | 2 230 – 78 384 | 9 069 | -60.79 |
|  | No IRS / LLIN | 10 969 | 5 456 – 27 548 | 3 949 | -26.47 |
| Future – RCP 4.5  (2036-2065) | No IRS / No LLIN | 19 290 | 9 781 – 124 712 |  |  |
|  | IRS / LLIN | 5 954 | 2 500 – 13 162 | 13 336 | -69.13 |
|  | IRS / No LLIN | 6 838 | 2 852 – 63 922 | 12 452 | -64.55 |
|  | No IRS / LLIN | 15 190 | 7 914 – 31 541 | 4 100 | -21.25 |
| Future – RCP 8.5  (2036-2065) | No IRS / No LLIN | 19 391 | 10 385 – 113 207 |  |  |
|  | IRS / LLIN | 6 293 | 2 780 – 14 471 | 13 098 | -67.55 |
|  | IRS / No LLIN | 6 987 | 3 294 – 50 096 | 12 404 | -63.97 |
|  | No IRS / LLIN | 16 033 | 9 158 – 34 615 | 3 358 | -17.32 |

LLIN = long-lasting insecticidal nets; IRS = Indoor residual spraying

**Table S5** - Equations of a generalized linear models (GLM) based on a negative binomial distribution used for predicting malaria risk in Uganda

| **Pooled model 1 (no interaction between environmental variables and interventions)** |
| --- |
| $\log(E\left( Y_{i} \right))= \beta_{0}+s\left( rainfall, df=2 \right)+s\left( minimum temperature, df=2 \right)+s\left( maximum temperature, df=2 \right)+s\left( \mathrm{humidity}, df=2 \right)+s\left( EVI, df=2 \right)+\beta_{1}\mathrm{IRS}+\beta_{2}\mathrm{LLIN}+\beta_{3}Average income+\beta_{4}site(df=5)+ offset(\log\left( Number of visits \right))$ |
| **Pooled model 1 (with interactions between environmental variables and interventions)** |
| $\log(E\left( Y_{i} \right))= \beta_{0}+s\left( rainfall, df=2 \right)+s\left( minimum temperature, df=2 \right)+s\left( maximum temperature, df=2 \right)+s\left( \mathrm{Humidity}, df=6 \right)+s\left( EVI, df=2 \right)++\beta_{1}\mathrm{IRS}+\beta_{2}\mathrm{LLIN}+\beta_{3}Average income+\beta_{4}site(df=5)+s\left( minimum temperature, df=2 \right)*IRS + s(EVI, df=2)*IRS + s(humidity, df=2)*IRS + s(rainfall, df=2)*LLIN + s(minimum temperature, df=2)*LLIN + s(maximum temperature, df=2)*LLIN + s(EVI, df=2)*LLIN+ offset(\log\left( Number of isits \right))$ |

E(Y) is the expected number of malaria cases, α is the intercept, s represents a smooth function using a smoothing spline.

GLMs are used when the response variable is not distributed normally and consist of non-negative integers. GLMs are made up of linear predictors that can be written as $y_{i}= \beta_{0}+ \beta_{1}x_{1i}+\ldots+\beta_{p}x_{p}+\varepsilon_{i}$, where the response $y_{i} , i=1, \ldots., n$ is modelled by a linear function of explanatory variables, plus an error term. The negative binomial distribution is used to deal with overdispersion in count data as it allows the conditional variance of the outcome variable to be greater than its conditional mean, which offers greater flexibility in model fitting.

**Table S6** - Summary of GLM negative binomial model without interaction^[[2]](#footnote-2)^

|  | **Malaria weekly cases** | | |
| --- | --- | --- | --- |
| *Predictors* | *Incidence Rate Ratios* | *95% CI* | *p* |
| IRS [Yes] | 0.37 | 0.33 – 0.40 | **< 2.20 e^-16^** |
| LLIN [Yes] | 0.65 | 0.62 – 0.68 | **< 2.20 e^-16^** |
| Average income | 9.99 e^-01^ | 9.99 e^-01^– 9.99 e^-01^ | **< 2.20 e^-16^** |
| Rainfall (mm) - 1 | 1.00 | 0.99 – 1.00 | 0.104 |
| Rainfall (mm) - 2 | 1.00 | 0.99 – 1.00 | 0.4339 |
| Minimum temperature (°C) - 1 | 1.40 | 1.27 – 1.55 | **1.23 e^-10^** |
| Minimum temperature (°C) - 2 | 0.81 | 0.74 – 0.90 | **0.000** |
| Maximum temperature (°C) - 1 | 0.70 | 0.64 – 0.77 | **5.50 e^-16^** |
| Maximum temperature (°C) - 2 | 1.14 | 1.08 – 1.21 | **4.96 e^-06^** |
| Specific humidity (kg.kg-1) - 1 | 6.88 e^07^ | 2.28 e^-07^ – 2.28 e^22^ | 0.278 |
| Specific humidity (kg.kg-1) - 2 | 9.99 e^14^ | 44.38 –2.07 e^28^ | **0.028** |
| EVI -1 | 42.8 | 15.63 – 117.46 | **3.07 e^-13^** |
| EVI - 2 | 0.15 | 0.04 – 0.48 | **0.001** |
| MRC^a^  Kamwezi | 0.24 | 0.17 – 0.34 | **< 2.20 e^-16^** |
| Kasambya | 1.01 | 0.79 – 1.29 | 0.934 |
| Kihihi | 0.83 | 0.63 – 1.09 | 0.175 |
| Nagongera | 0.75 | 0.59 – 0.95 | **0.019** |
| Walukuba | 0.28 | 0.24 – 0.33 | **< 2.20 e^-16^** |
| Observations  R^2^ Nagelkerke | 2787  0.661 | | |
|  |  | | |

^a^Aduku is the reference

LLIN = long-lasting insecticidal nets; IRS = Indoor residual spraying; EVI = enhanced vegetation index

**Table S7** - Summary of GLM negative binomial model with interaction interactions ^[[3]](#footnote-3)^

|  | **Malaria weekly cases** | | |
| --- | --- | --- | --- |
| *Predictors* | *Incidence Rate Ratios* | *95% CI* | *p* |
| IRS [Yes] | 0.89 | 0.00 – 1.57 | >0.9 |
| LLIN [Yes] | 0.00 | 0.00 – 0.00 | <0.001 |
| Average income | 1.00 | 1.00 – 1.00 | <0.001 |
| Rainfall (mm) - 1 | 1.00 | 1.00 – 1.00 | <0.001 |
| Rainfall (mm) - 2 | 1.00 | 1.00 – 1.00 | 0.027 |
| Minimum temperature (°C) - 1 | 1.75 | 1.59 – 1.96 | **<0.001** |
| Minimum temperature (°C) - 2 | 0.68 | 0.61 – 0.77 | **<0.001** |
| Maximum temperature (°C) - 1 | 0.57 | 0.52 – 0.63 | **<0.001** |
| Maximum temperature(°C) - 2 | 1.25 | 1.18 – 1.34 | **<0.001** |
| Specific humidity (kg.kg-1) - 1 | 0.00 | 0.00 – 0.00 | <0.001 |
| Specific humidity (kg.kg-1) - 2 | 1.01e^57^ | 1.54e^43^ – 7.65e^70^ | **<0.001** |
| EVI -1 | 4.55 | 0.95 – 21.4 | 0.060 |
| EVI - 2 | 0.45 | 0.08 – 2.47 | 0.4 |
| MRC^a^  Kamwezi | 0.33 | 0.23 – 0.47 | **<0.001** |
| Kasambya | 1.21 | 0.95 – 1.54 | 0.13 |
| Kihihi | 1.11 | 0.85 – 1.45 | 0.4 |
| Nagongera | 0.99 | 0.77 – 1.27 | >0.9 |
| Walukuba | 0.29 | 0.25 – 0.35 | **<0.001** |
| IRS* Minimum temperature - 1 | 0.71 | 0.45 – 1.10 | 0.2 |
| IRS* Minimum temperature - 2 | 1.52 | 1.06 – 2.19 | 0.049 |
| IRS*EVI -1 | 1.33 | 0.19 – 9.41 | 0.8 |
| IRS*EVI -2 | 31.5 | 2.15 – 474 | **0.013** |
| IRS*humidity -1 | 3.80e^166^ | 7.32e^123^ –1.14e^209^ | **<0.001** |
| IRS*humidity - 2 | 0.00 | 0.00 – 0.00 | <0.001 |
| LLIN*Rainfall - 1 | 1.00 | 1.00 – 1.00 | <0.001 |
| LLIN*Rainfall - 2 | 1.00 | 1.00 – 1.00 | <0.001 |
| LLIN*Minimum temperature - 1 | 0.97 | 0.84 – 1.14 | 0.7 |
| LLIN*Minimum temperature - 2 | 0.99 | 0.86 – 1.14 | 0.9 |
| LLIN*Maximum temperature - 1 | 1.39 | 1.22 – 1.57 | **<0.001** |
| LLIN* Maximum temperature - 2 | 0.87 | 0.79 – 0.96 | **0.003** |
| LLIN*EVI - 1 | 20.3 | 2.90 – 143 | **0.003** |
| LLIN*EVI - 2 | 0.24 | 0.02 – 2.40 | 0.2 |
| Observations  R^2^ Nagelkerke | 2787  0.750 | | |
|  |  | | |

^a^Aduku is the reference center

LLIN = long-lasting insecticidal nets; IRS = Indoor residual spraying; EVI = enhanced vegetation index

**Table S8** - Calculation of over-optimism to assess the risk of overfitting

| **Pooled model 1 (no interaction between environmental variables and interventions)** | |
| --- | --- |
| RMSE | 41.7081 |
| Optimism | 0.0048 |
| Optimism corrected RMSE | 41.713 |
| **Pooled model 1 (with interactions between environmental variables and interventions)** | |
| RMSE | 39.686 |
| Optimism | 0.223 |
| Optimism corrected RMSE | 39.908 |

RMSE : root mean square error

Procedure for calculating optimism with cross-validation:

1. Randomly divide the data into 10 parts
2. Estimate the model on 9/10 and calculate the RMSE on the remaining 1/10
3. Repeat previous step for every 1/10
4. Repeat this process (steps 1 to 3) 100 times

Optimism = (average RMSE over the 1000 values) - RMSE of the original model

Optimism corrected RMSE = original RMSE + optimism

**
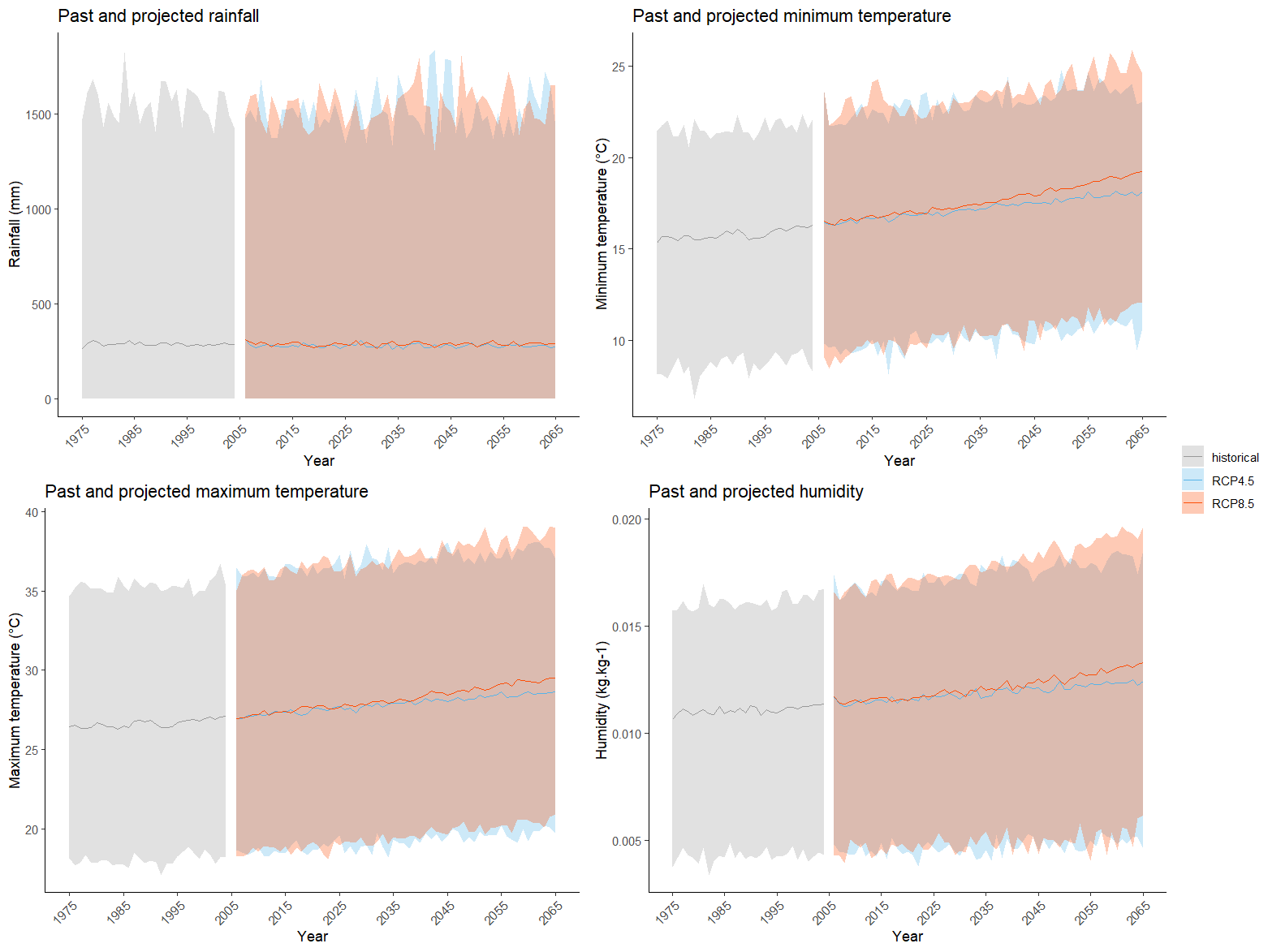
Figure S1.** Annual trends (based on weekly data) of environmental variables for the historical (1975-2004) and future periods (2036-2065)


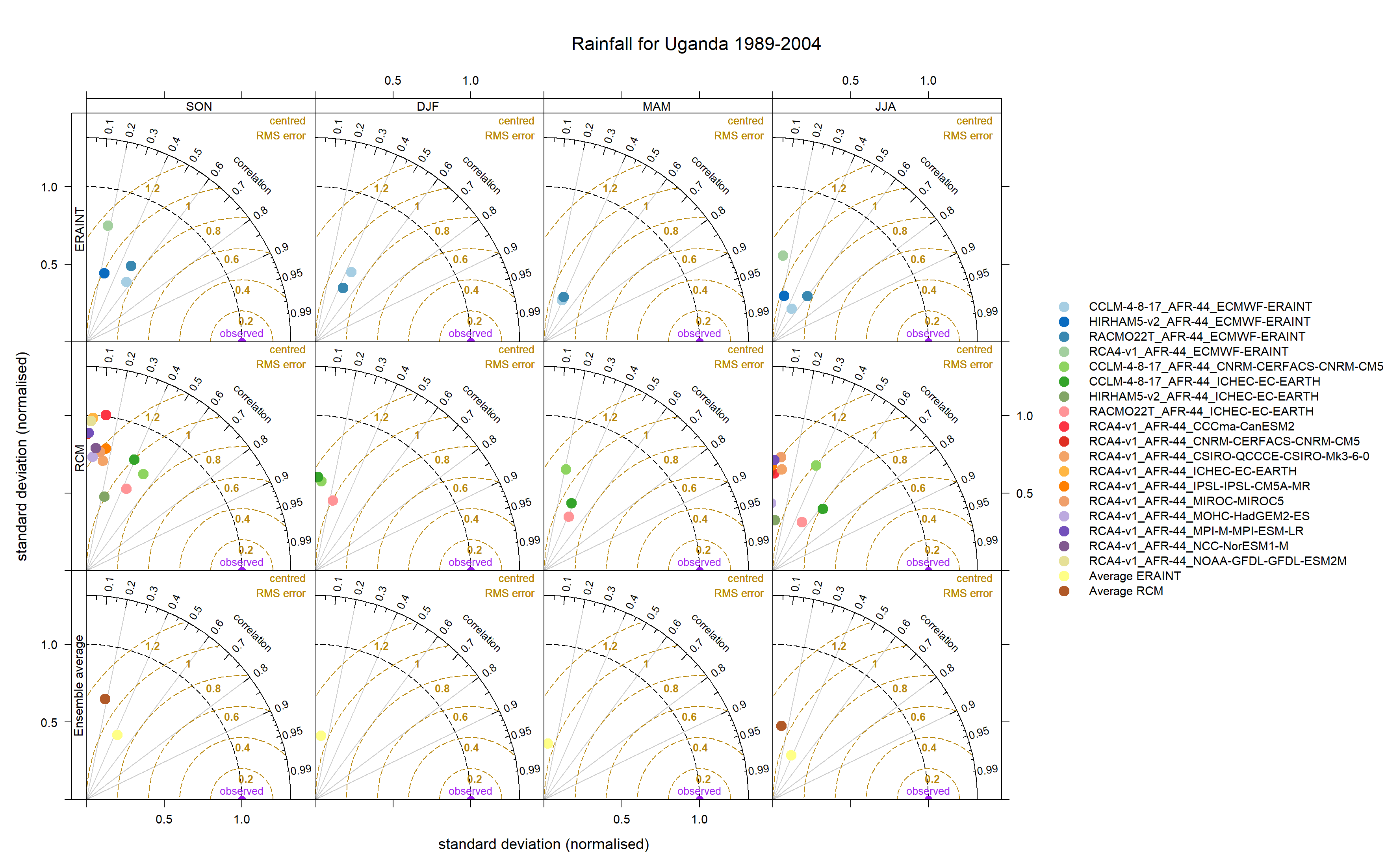
**Figure S2.** Taylor diagrams showing seasonal precipitation for Uganda from 1989 to 2004

Neither the GCM-driven RCMs and ERA-interim driven RCMs nor the ensemble average show a good correlation with the observed data (*R* < 0.6). Comparing normalized standardized standard deviations reveals that almost all GCM-driven RCMs and and ERA-interim driven RCMs exhibit an underestimation of the spatial variability of precipitations, implying that all models exhibit a lower seasonal fluctuation compared to the observed data. It should be noted that GCMs-driven RCMs, unlike ERA-interim driven RCMs, are not designed to analyze high-frequency variability (such as seasonal variability presented here) but to describe the global characteristics of the climate. This could explain the low performance of these models to simulate rainfall.


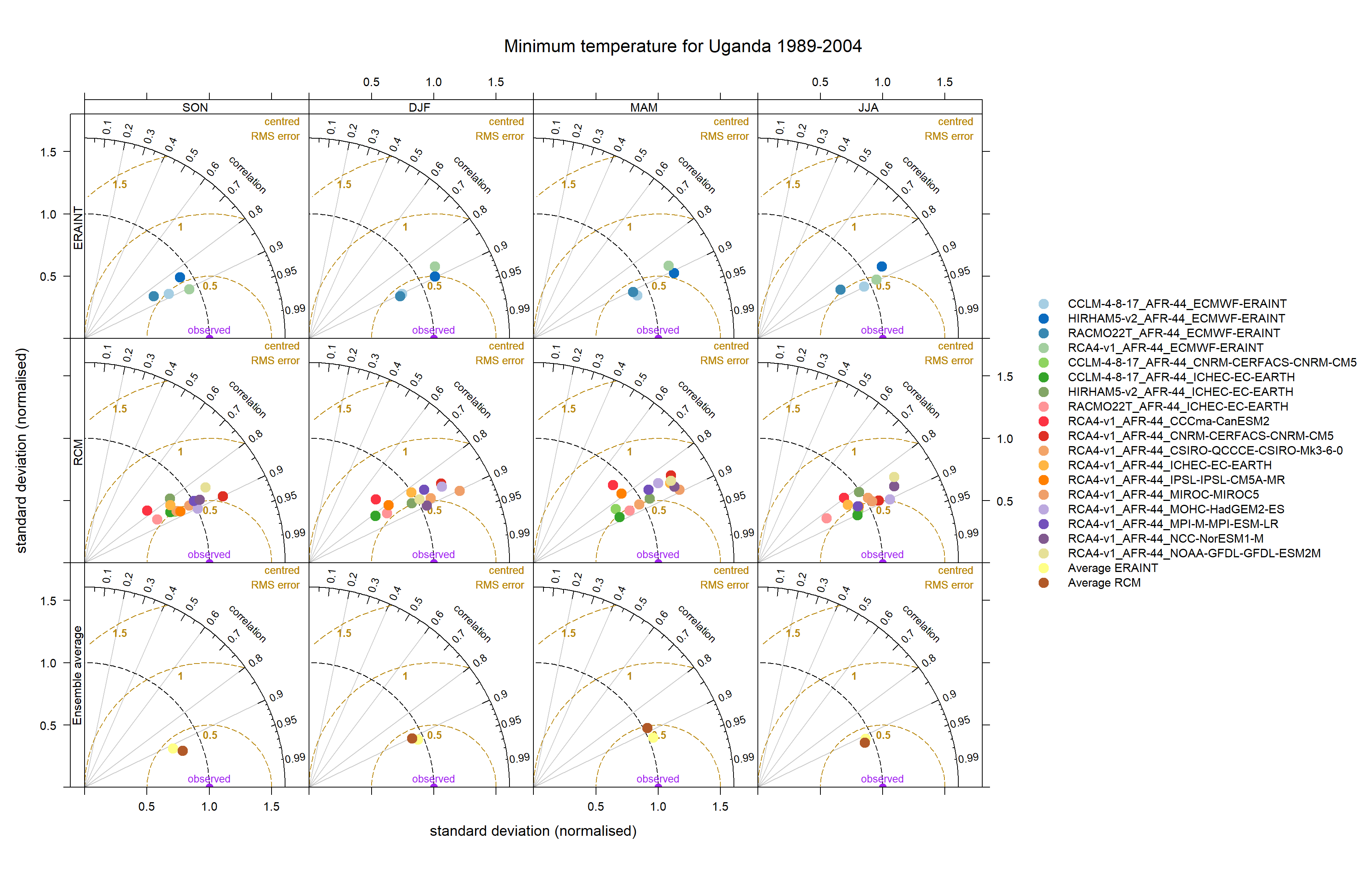
**Figure S3.** Taylor diagrams showing seasonal minimum temperature for Uganda from 1989 to 2004

Both GCM-driven RCMs and ERA-interim driven RCMs exhibit relatively high Pearson's Correlation coefficients (*R* > 0.8) and a low centred RMSE (+/- 0.5). Half of GCM-driven RCMs and ERA-interim-driven RCMs tended to overestimate maximum temperature variability as they are located above the standard deviation of the observed data. Given that GCMs-driven RCMs are not designed to analyze high-frequency variability (such as seasonal variability presented here), the good performance observed of these models is probably due to chance.

**
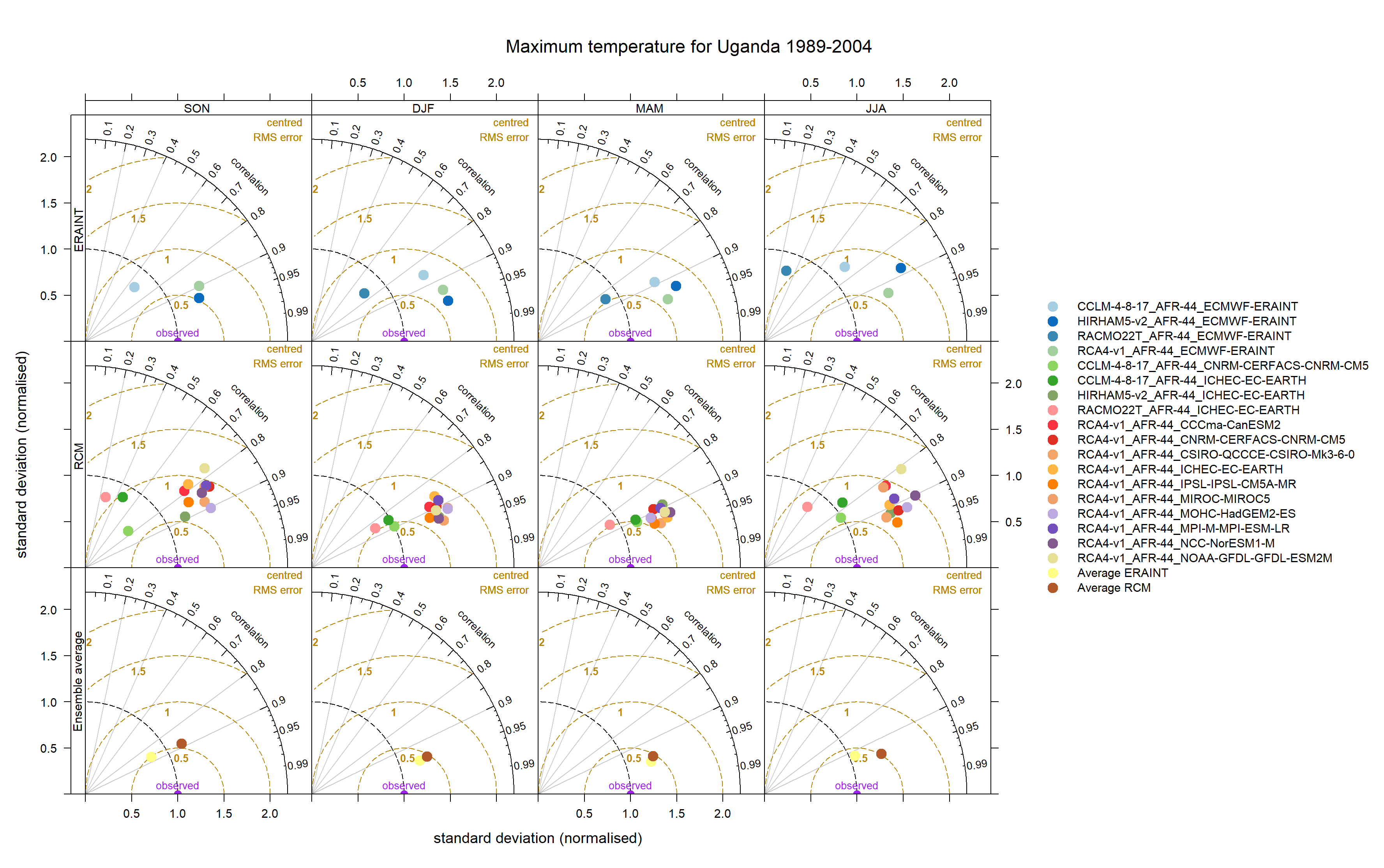
Figure S4.** Taylor diagrams showing seasonal maximum temperature for Uganda from 1989 to 2004

Both GCM-driven RCMs and ERA-interim driven RCMs exhibit relatively high Pearson's Correlation coefficients (*R* > 0.8) and a low centred RMSE (+/- 0.5). Half of GCM-driven RCMs and ERA-interim-driven RCMs tended to overestimate maximum temperature variability as they are located above the standard deviation of the observed data. Given that GCMs-driven RCMs are not designed to analyze high-frequency variability (such as seasonal variability as presented here), the good performance observed of these models is probably due to chance.

**
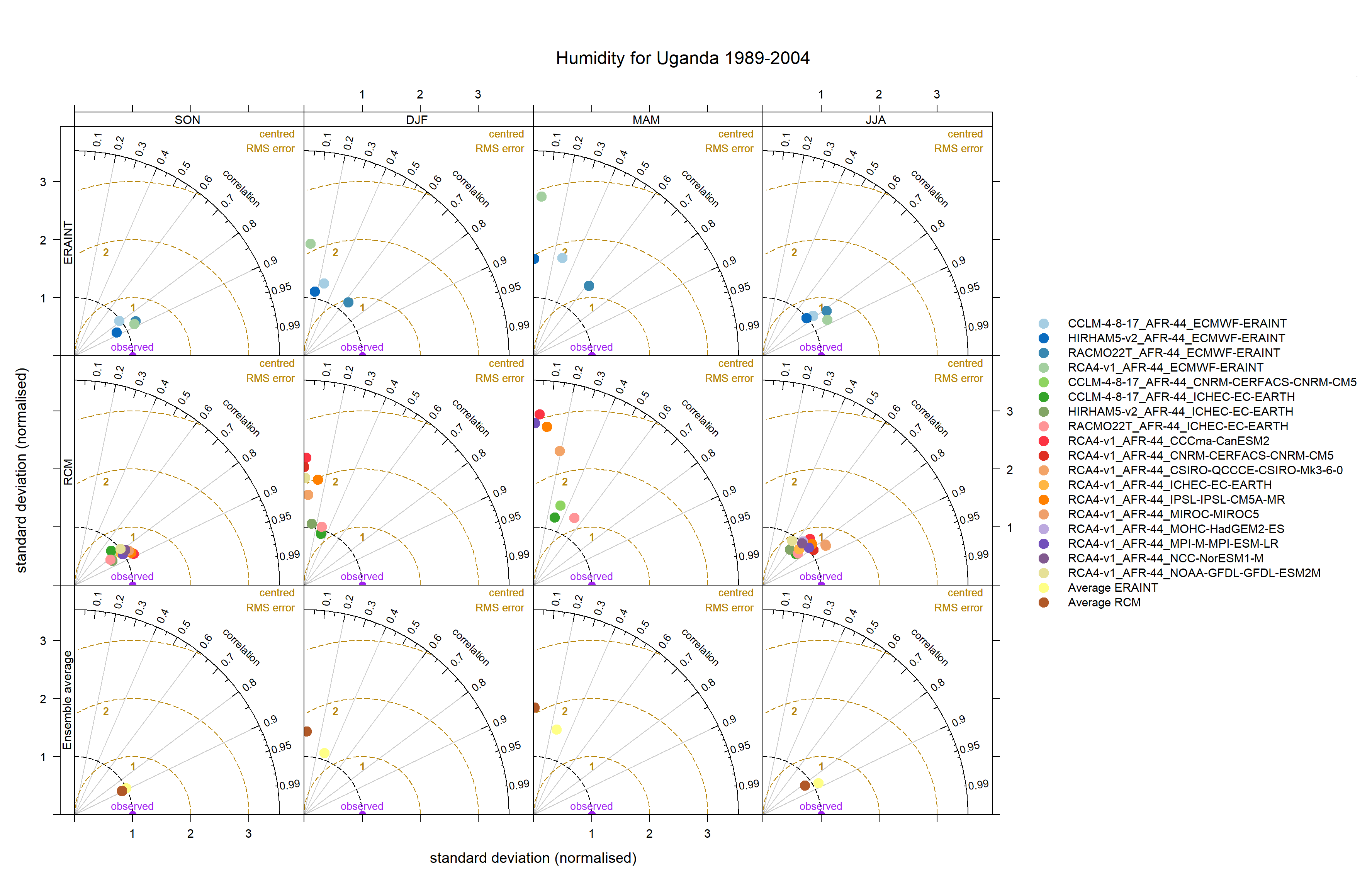
Figure S5.** Taylor diagrams showing seasonal specific humidity for Uganda from 1989 to 2004

Models performances was seasonally variable, with both GCM-driven RCMs and ERA-interim driven RCMs showing better performance overall (i.e., higher Pearson’s Correlation coefficients, smaller centred RMSE, and standard deviation closer to observations) for periods between September-November and June-August. It should be noted that GCMs-driven RCMs, unlike ERA-interim driven RCMs, are not designed to analyze high-frequency variability (such as seasonal variability presented here).


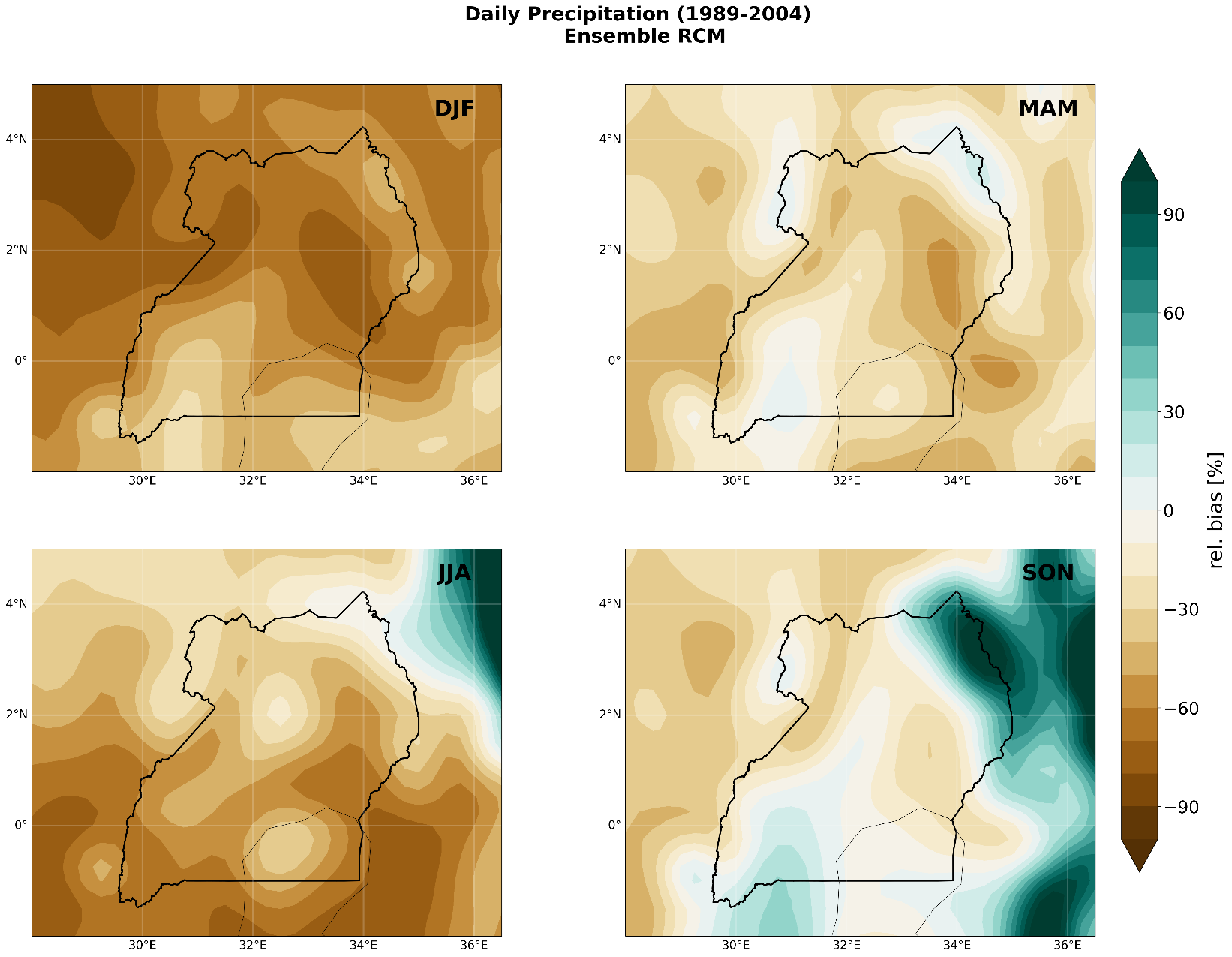


**Figure S6** - Comparison of seasonal relative biases of rainfall over Uganda from 1989 to 2004

Relatively high dry biases (>50%) are observed over the northern part of Uganda between December and February (DJF) and more in the southern part between June and August (JJA), while a wet bias (>70%) is present in the northeastern extremity of the country between September and November (SON). The March to May (MAM) and September to November (SON) periods have the least bias. Wet biases indicate that precipitation simulated by GCM-driven RCMs is higher than the precipitation observed by ERA5, while dry biases indicate that precipitation simulated by GCM-driven RCMs is lower than that of ERA5

**
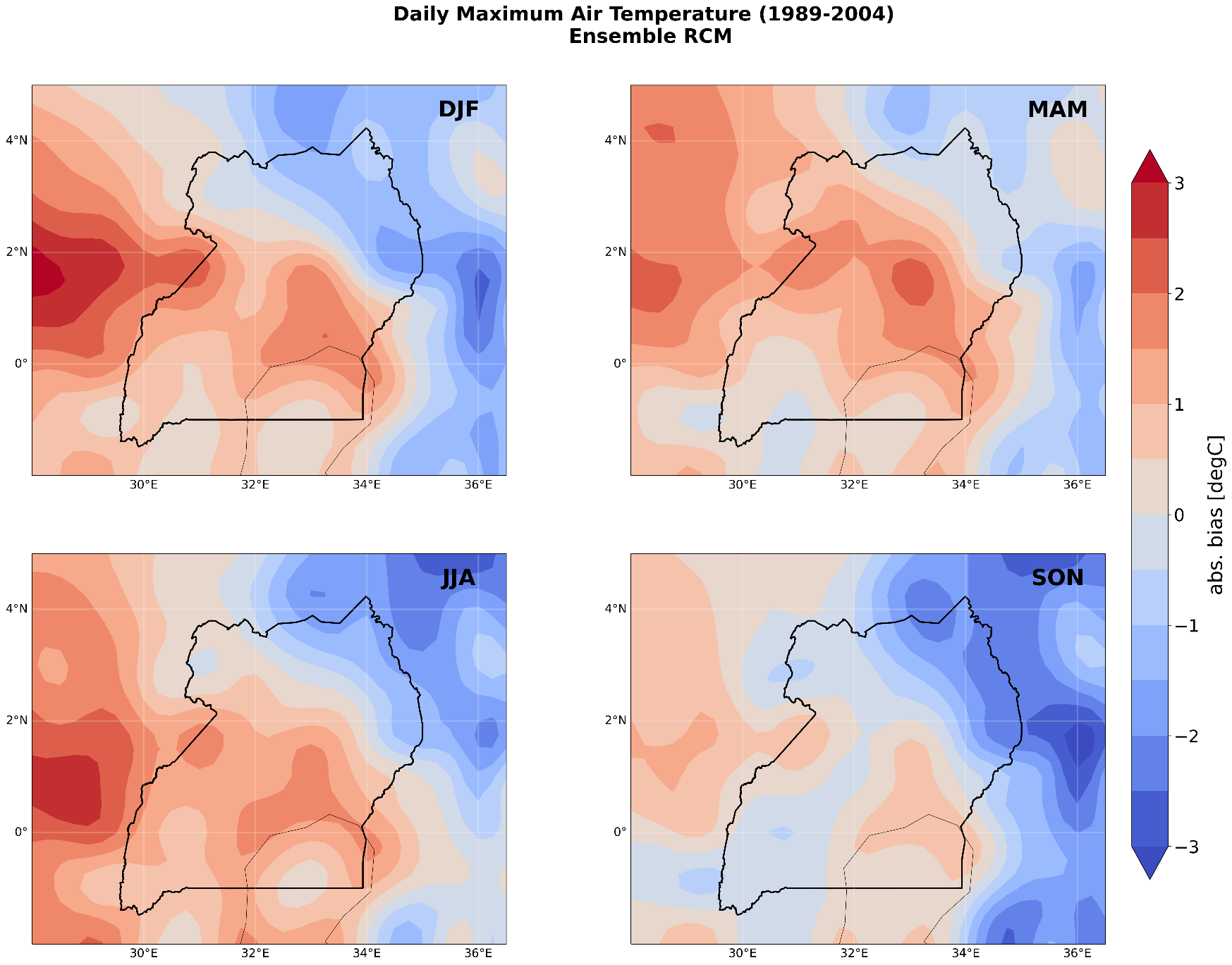
Figure S7** – Comparison of seasonal absolute biases of maximum temperature over Uganda from 1989 to 2004

The east-central part of Uganda has a greater warm bias (between +1.5°C and +2°C) for most seasons, while a cold bias (-0.5°C to -2°C) is present in the north-eastern extremity of the country. A warm bias indicates that the maximum temperatures simulated by the GCM driven RCM are higher than the maximum temperatures observed by ERA5, and a cold bias indicates that the maximum temperatures simulated by GCM driven RCM are lower than those observed by ERA5

**
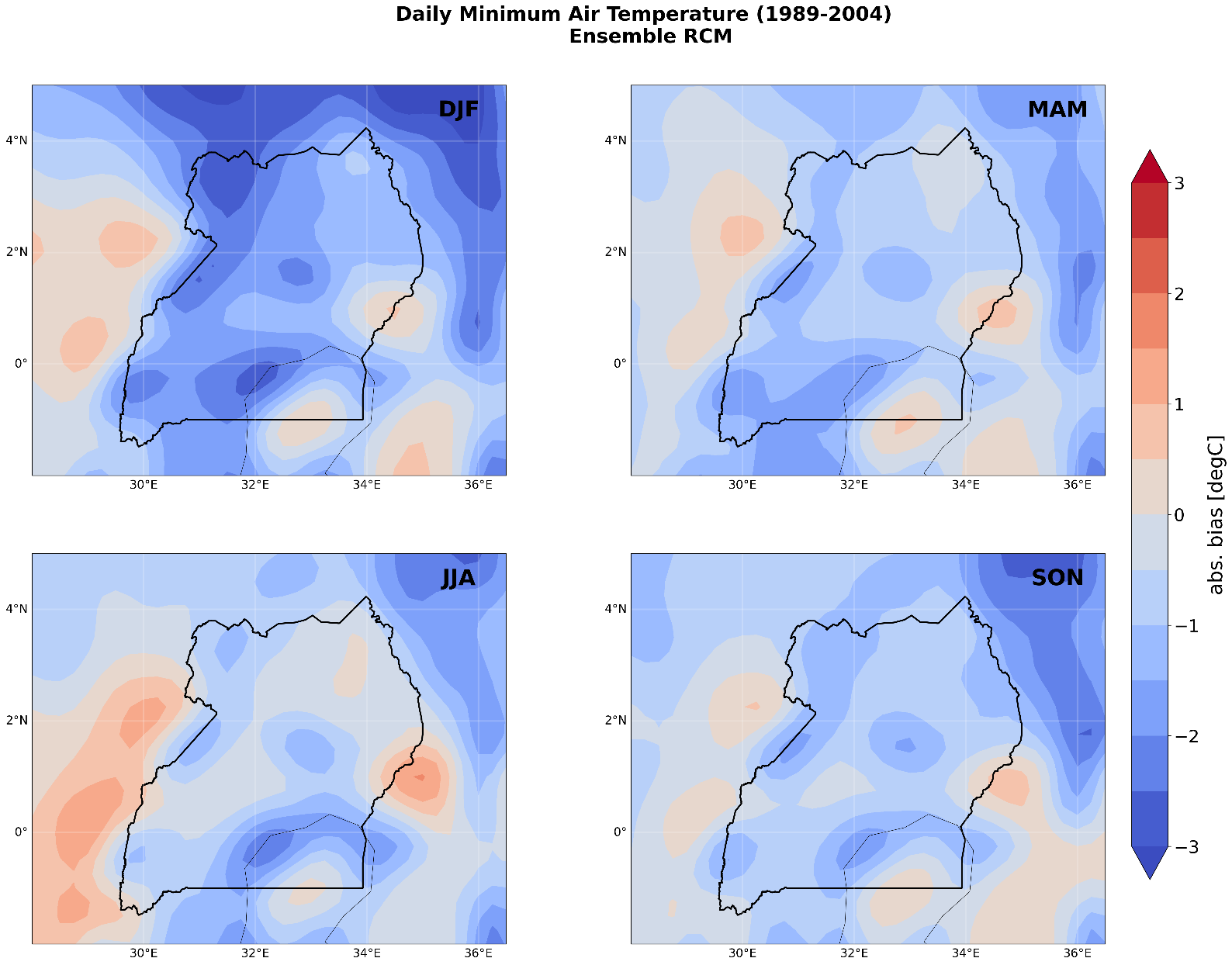
Figure S8** - Comparison of seasonal absolute biases of minimum temperature over Uganda from 1989 to 2004

The majority of biases observed across Uganda for minimum temperatures are cold biases, the largest of which (up to -2.5°C) are observed for the period December to February (DJF). A cold bias indicates that the minimum temperatures simulated by the GCM driven RCM are lower than those observed by ERA5

**
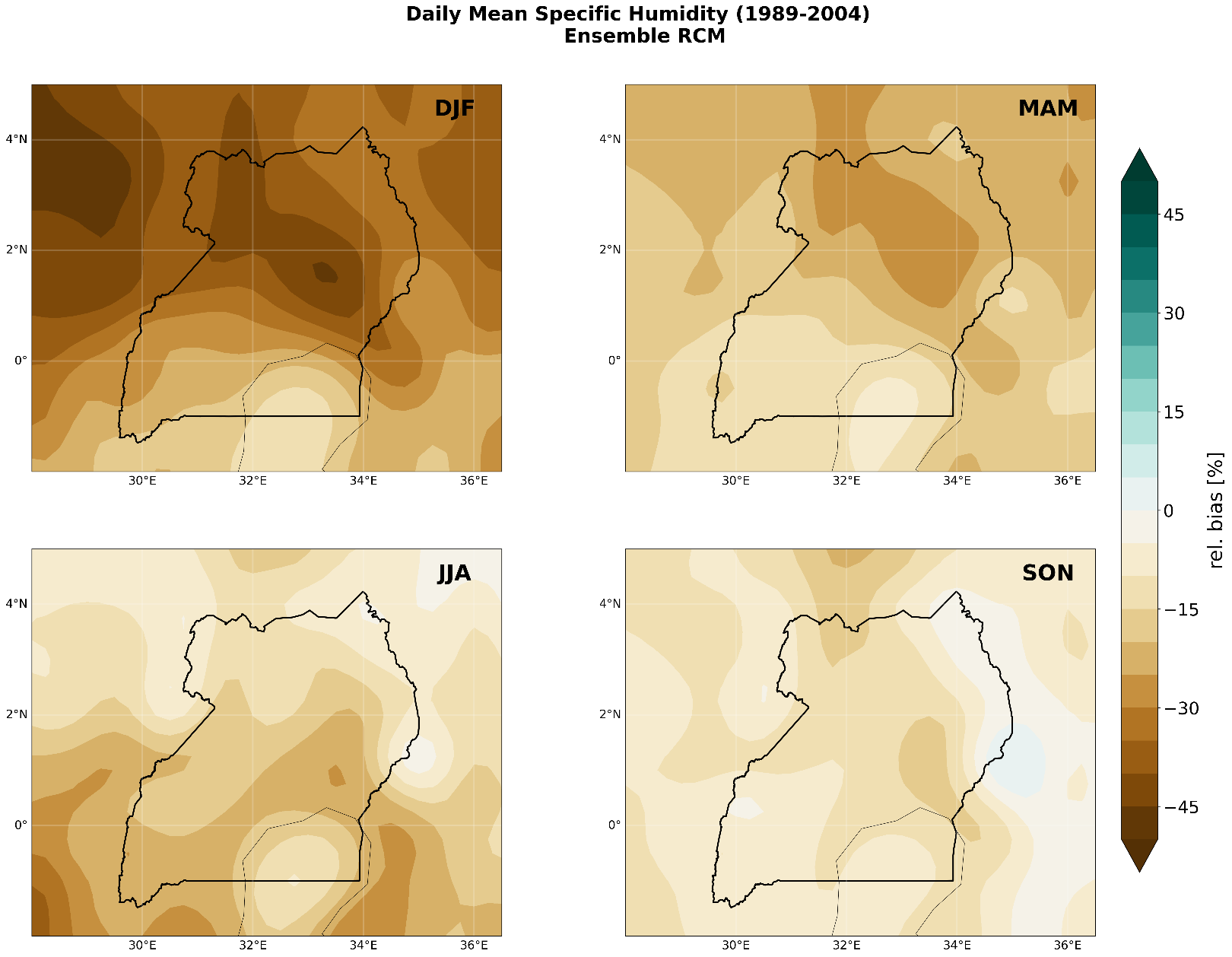
Figure S9** - Comparison of seasonal relative biases of humidity over Uganda from 1989 to 2004

For specific humidity, only dry biases are observed for the different seasons. A relatively significant dry bias (>40% and more) is present over the central and northwestern part of Uganda between December and February (DJF). Low biases (between -5 and -15%) are observed across Uganda between September and November (SON). The dry biases indicate that the specific humidity simulated by the GCM-driven RCMs is lower than the humidity observed by ERA5


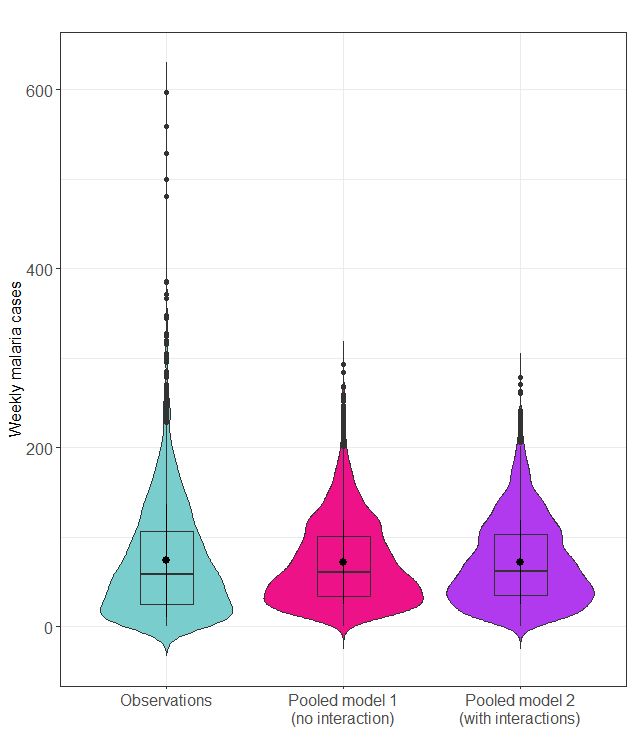
**Figure S10.** Comparison of observed and predicted malaria weekly cases over 2010-2018 from negative binomial GLMs; The mean of the distributions is represented by the black dots (N=53 weeks*6 regions *9 years)


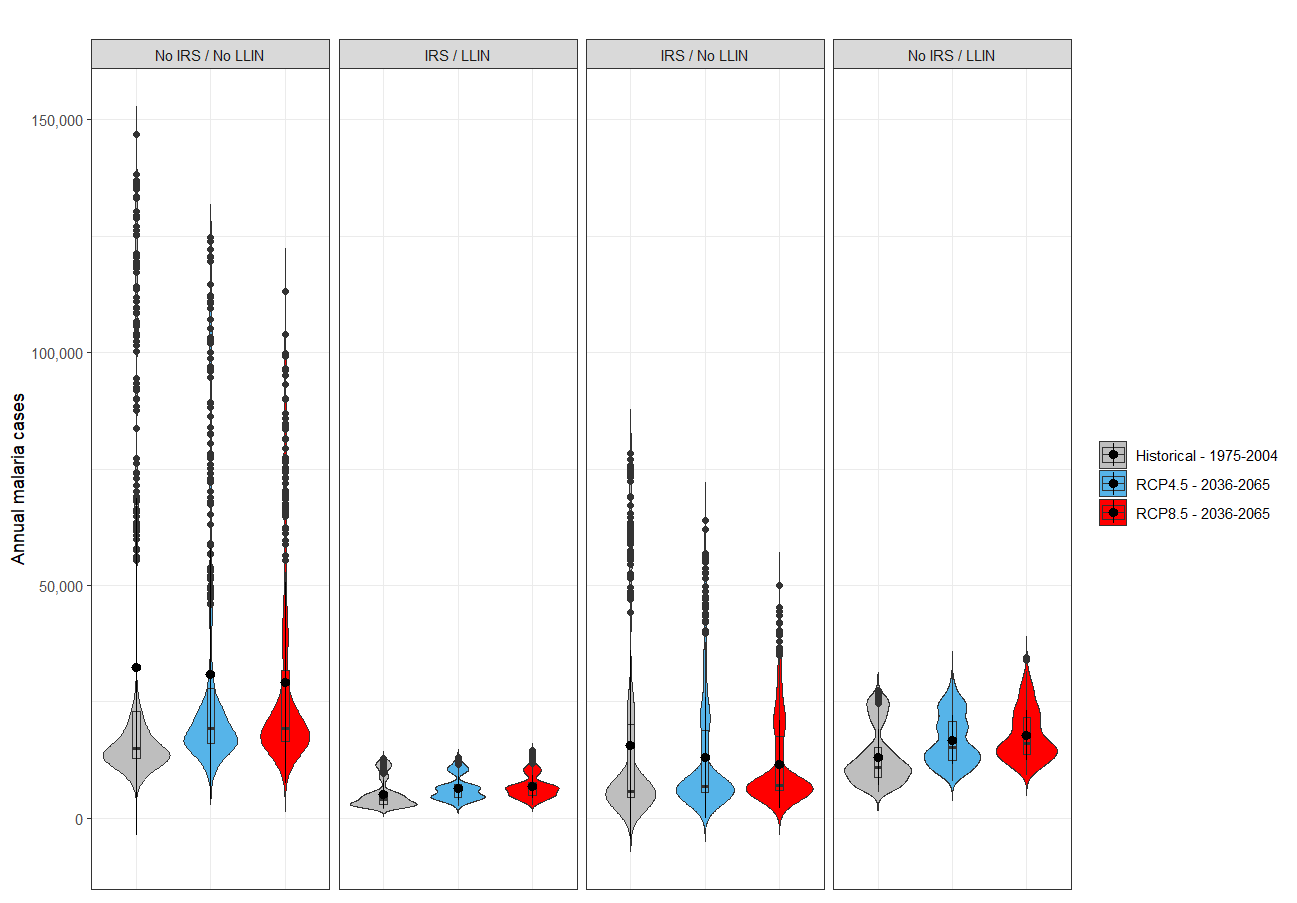


**Figure S11.** Distribution of annual sum of weekly malaria cases predicted for the past (1975-2004) and the future (2036-2065) periods; Predictions from the negative binomial GLM considering significant interactions between environmental variables and vector control interventions; The mean of the distributions is represented by the black dots; (N=30 years*14 climate models*4 interventions*3 periods) ; LLIN = long-lasting insecticidal nets; IRS = Indoor residual spraying


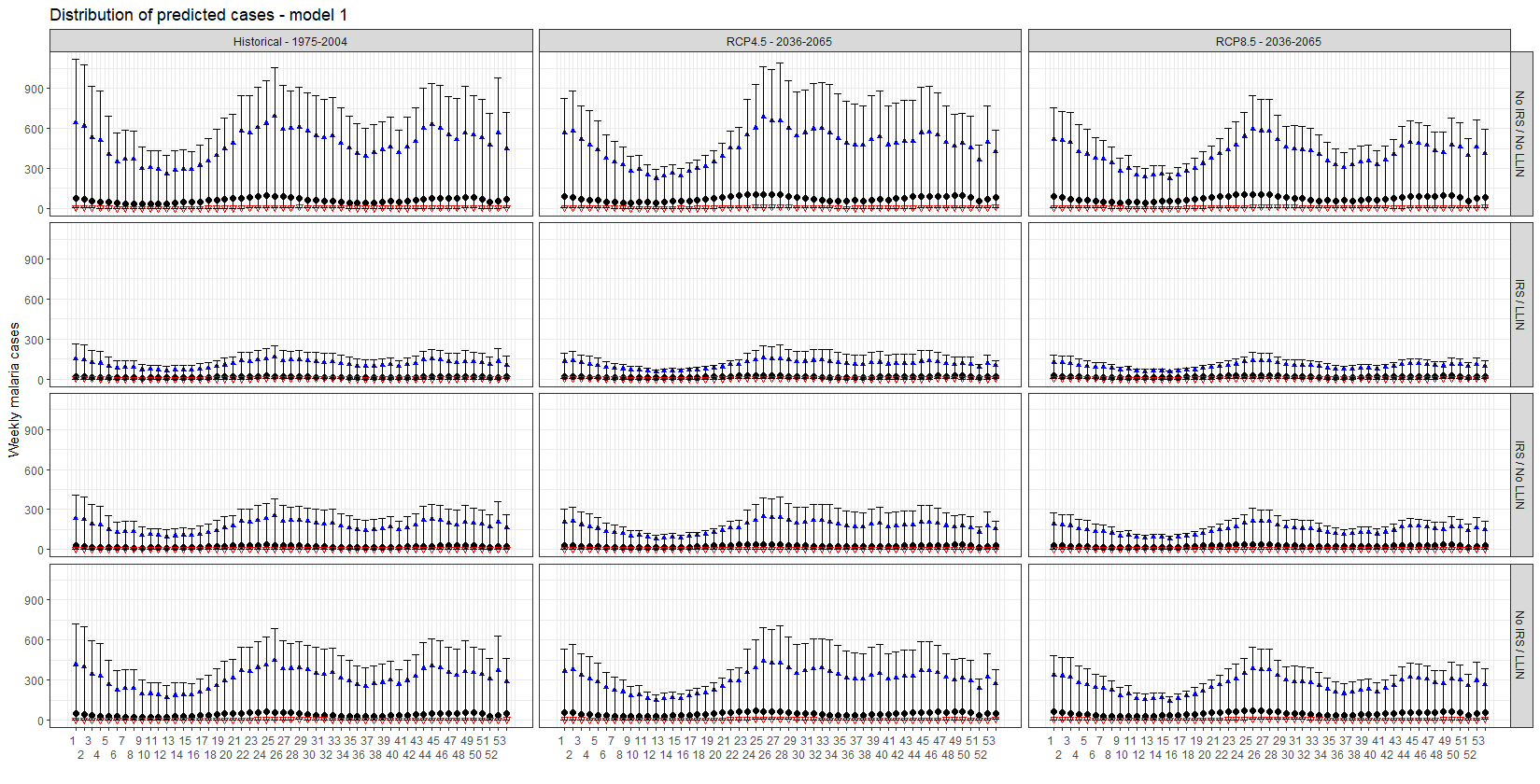
**Figure S12.** Distribution of weekly malaria cases predicted for the past (1975-2004) and the future (2036-2065) periods for the model with no interaction; The median, the maximal and minimal prediction are represented by the black dot, the blue triangle and the red triangle, respectively; the 95% CI of the maximum and minimum predicted cases is presented; (N= 53 weeks*6 health centers*14 climate models*4 interventions*30 years*3 periods) ; LLIN = long-lasting insecticidal nets; IRS = Indoor residual spraying

**
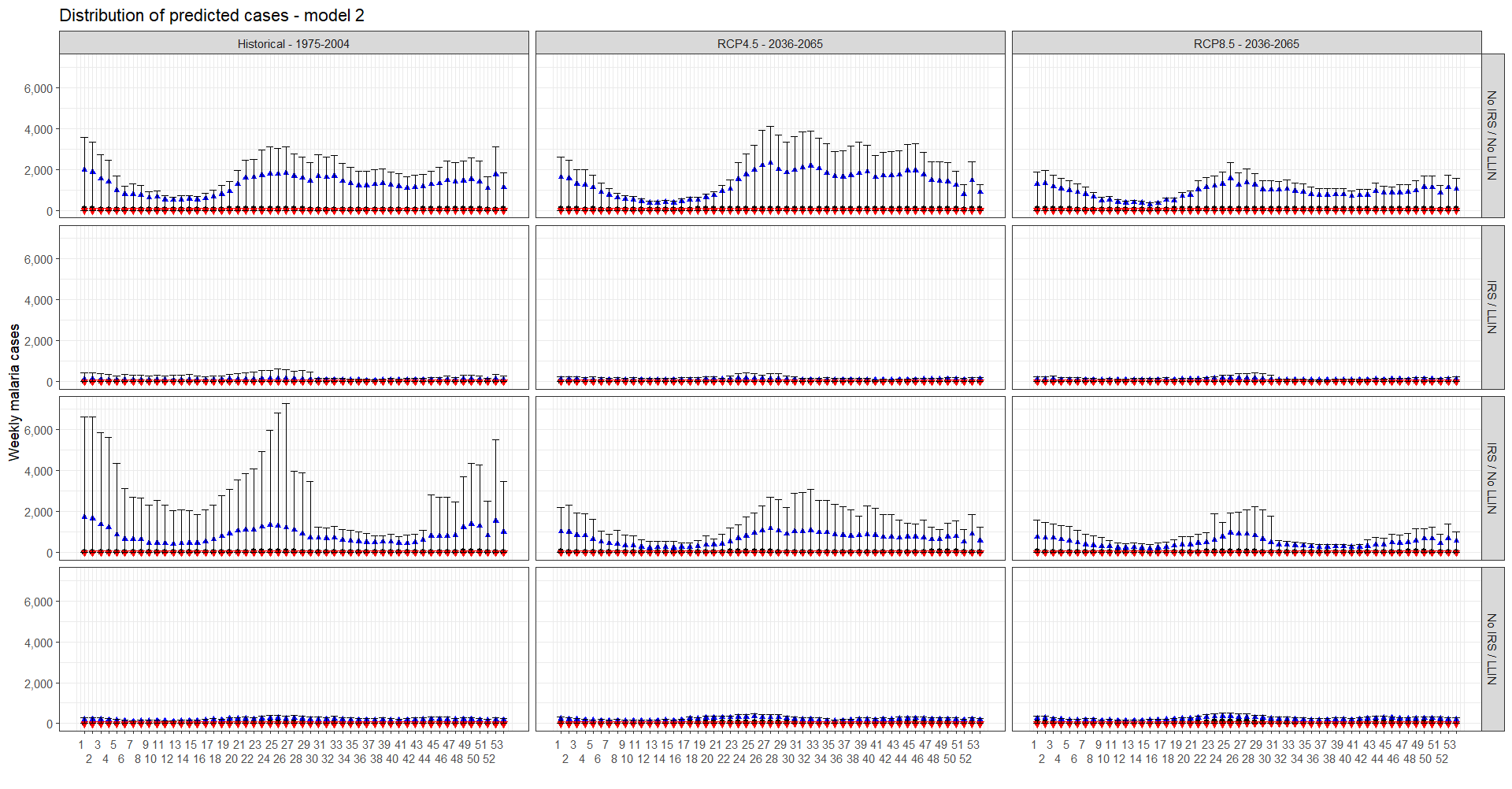
Figure S13.** Distribution of weekly malaria cases predicted for the past (1975-2004) and the future (2036-2065) periods for the model with interaction; The median, the maximal and minimal prediction are represented by the black dot, the blue triangle and the red triangle, respectively; the 95% CI of the maximum and minimum predicted case is presented; (N= 53 weeks*6 health centers*14 climate models*4 interventions*30 years*3 periods) ; LLIN = long-lasting insecticidal nets; IRS = Indoor residual spraying

1. Data are described in Sadoine, M. L., Smargiassi, A., Liu, Y., Gachon, P., Fournier, M., Dueymes, G., ... & Zinszer, K. (2023). Differential Influence of Environmental Factors on Malaria Due to Vector Control Interventions in Uganda. International Journal of Environmental Research and Public Health, 20(22), 7042. [↑](#footnote-ref-1)
2. Data used for this model are described in Sadoine, M. L., Smargiassi, A., Liu, Y., Gachon, P., Fournier, M., Dueymes, G., ... & Zinszer, K. (2023). Differential Influence of Environmental Factors on Malaria Due to Vector Control Interventions in Uganda. International Journal of Environmental Research and Public Health, 20(22), 7042. [↑](#footnote-ref-2)
3. Data used for this model are described in Sadoine, M. L., Smargiassi, A., Liu, Y., Gachon, P., Fournier, M., Dueymes, G., ... & Zinszer, K. (2023). Differential Influence of Environmental Factors on Malaria Due to Vector Control Interventions in Uganda. International Journal of Environmental Research and Public Health, 20(22), 7042. [↑](#footnote-ref-3)
