# Supplementary material for: Predicting malaria risk considering vector control interventions under climate change scenarios
Source: Sci Rep. 2024 Jan 29;14:2430. doi: 10.1038/s41598-024-52724-x (PMC10824718; doi:10.1038/s41598-024-52724-x)
Supplement: Supplementary file 2 — Supplementary Information 2. [file 41598_2024_52724_MOESM2_ESM.docx]

**Supplementary material – file 2**

Projecting malaria risk considering vector control interventions under climate change scenarios -

Margaux L. Sadoine, Kate Zinszer, Ying Liu, Philippe Gachon, Michel Fournier, Guillaume Dueymes, Grant Dorsey, Ana Llerena, Jane Frances Namuganga, Bouchra Nasri, and Audrey Smargiassi

***Climate models and regression model assessment***

Temporal biases of climate models evaluated using Taylor diagrams for the period 1989-2004 revealed varied performance levels depending on the weather variables. The climate models (both GCM-driven RCMs and ERA-interim driven RCMs) poorly reproduced the temporal variability of precipitation, showing a constant underestimation of the seasonal fluctuation compared to the observed data. In contrast, projected temperatures showed good correlations and reproducibility of the temporal variability of the observed data. Further details and comprehensive results, including Taylor diagram plots, are provided in the Supplementary Figures S2 to S5. Results of spatial bias analyzes suggested that precipitation and specific humidity simulated by GCM driven RCMs were lower than the observed data for the period 1989-2004 for most seasons over a large part of Uganda. Minimum temperatures simulated by the GCM driven RCMs ensemble were mostly lower than the minimum temperatures observed, but in a geographically variable manner depending on the season, while the spatial biases of the maximum temperatures were mixed (the simulations by the GCM driven RCMs being lower than the observations in the extreme north-east of the country, and higher in the southern part of Uganda). Further details and comprehensive results, including maps of spatial biases, are provided in the Supplementary Figures S6 to S9.

The comparison of the distributions between the weekly observed and predicted malaria cases over 2010-2018 is shown in Figure S6. The mean and median of predicted cases were relatively similar to the observed cases (mean observed vs predicted cases: 73.3 vs 74.1; median observed vs predicted cases: 58.0 vs 65.4); although the model with interactions predicted a slightly higher median number of cases than observed (model with interaction: 68.9). However, results showed a smaller distribution of predicted cases, i.e., the maximum value of the weekly predicted cases was lower than the maximum value of the observed cases, and the minimum predicted value was higher than the minimum of observed cases. There was also a wider range of certain predicted values compared to observed cases.

The error associated with the prediction was estimated at approximately 42 cases for the model without interaction (RMSE = 41.7) and 40 for the model with interactions (RMSE = 39.7) (Table S8); mean observed cases were ~73. However, over-optimism (based on cross-validation) was very low for both models (<1) indicating that overfitting is negligible and a similar accuracy should be obtained on a new dataset.
